# Practice leadership development in intellectual disability services: A qualitative study of practice leaders’ experiences

**DOI:** 10.1371/journal.pone.0351893

**Published:** 2026-11-03

**Authors:** Owen Doody, Maeve O’Halloran, Eileen Carey, Marie Kilduff, Ruth Ryan

**Affiliations:** 1 Centre for Implementation Research at UL, Health Research Institute, School of Nursing and Midwifery, University of Limerick, Limerick, Ireland; 2 Intellectual Disability Services, Clare, Ireland; 3 Health Research Institute, School of Nursing and Midwifery, University of Limerick, Limerick, Ireland; 4 National Clinical Leadership Centre for Nursing and Midwifery, Health Service Executive, Dublin, Ireland; 5 School of Nursing and Midwifery, University of Limerick, Limerick, Ireland; Washington University in St Louis, Bursky School of Public Health, UNITED STATES OF AMERICA

## Abstract

**Background:**

Leadership in intellectual disability services is recognised as having a key role in achieving positive outcomes for people with intellectual disabilities, service quality and workforce wellbeing. However, limited research has explored how leadership capability is developed and enacted by practice leaders within everyday service contexts.

**Methods:**

This study reports the qualitative findings from a national mixed‑methods evaluation of a bespoke Clinical Leadership Programme for practice leaders in intellectual disability services in Ireland. Following ethical approval, individual semi‑structured interviews were conducted with fourteen participants who completed the programme. Data were analysed using inductive content analysis.

**Results:**

Participants described leadership development as a transformative process involving shifts in professional identity, confidence and legitimacy rather than the acquisition of discrete leadership skills. Reflection emerged as an important mechanism underpinning leadership development, supporting participants to move from reactive and task‑focused approaches towards more intentional, relational and ethically grounded leadership practice. Participants described the programme as reducing professional isolation and facilitating peer learning relationships that promoted psychological safety and supported learning within a community of practice. Participants also described changes in leadership enactment characterised by greater staff engagement, relational conflict management and the extension of person-centred values to staff teams. Re-engagement with education was viewed as an important outcome, reframing learning as an ongoing component of leadership development and professional growth. However, participants identified systemic challenges, including administrative burden, role overload and compliance-driven organisational cultures, as constraining the enactment of leadership in practice.

**Conclusion:**

Leadership development in intellectual disability services was described as an identity-based, relational and contextually constrained process. Reflective, practice-based and context-specific leadership programmes may support leadership capacity development; however, participants emphasised that the enactment of leadership in practice also requires supportive organisational structures and cultures.

## Introduction

Leadership within intellectual disability services has been repeatedly identified as a critical determinant of service quality, staff wellbeing and outcomes for people with intellectual disabilities. Despite this recognition, leadership in this field remains under‑researched, poorly theorised in practice contexts and frequently constrained by organisational and regulatory pressures [1–3]. Intellectual disability service staff, particularly those in frontline and middle-management roles, exercise leadership within increasingly complex environments characterised by heightened accountability, regulatory oversight and ongoing demands to deliver person-centred, rights-based care. Practice leadership has emerged as a conceptually distinct and practice‑grounded approach to leadership within intellectual disability services [4]. Unlike traditional hierarchical or managerial models, practice leadership is enacted close to the point of care and is concerned with shaping staff practice through modelling, coaching, feedback, supervision and relational engagement [5,6]. Empirical studies have demonstrated associations between effective practice leadership and improved staff experiences, reduced stress, enhanced active support and better service user engagement [1,7,8]. However, most of this literature has focused on observable leadership behaviours or staff‑rated leadership measures, with less attention given to the lived experience of leaders themselves or to leadership development as a process of identity formation, reflection and relational change.

Within scholarship, leadership is increasingly understood as relational, distributed and ethically grounded, particularly in care contexts involving vulnerability, power imbalances and sustained emotional labour [9–11]. Intellectual disability practice leaders are uniquely positioned at the intersection of health, social care, human rights and advocacy, requiring leaders who can navigate compliance demands while sustaining person‑centred cultures. Yet intellectual disability practice leaders frequently report professional isolation, limited access to tailored leadership development and insufficient recognition of the complexity of their leadership roles [3,12]. In response to these challenges, a national Clinical Leadership Programme for Persons in Charge (PICs) working in intellectual disability services in Ireland was designed, delivered and evaluated through a collaboration between the National Clinical Leadership Centre for Nursing and Midwifery and the University of Limerick. The programme was underpinned by principles of practice leadership, reflective practice, experience‑based co‑design and a rights‑based understanding of care. The programme’s design, implementation and mixed-methods evaluation have been reported previously by Doody et al. [13]. That evaluation demonstrated significant improvements in leadership confidence, perceived competence and career development indicators. The present study extends those findings through a stand-alone qualitative analysis exploring participants lived experiences of leadership development and the processes through which they perceived change to occur.

Quantitative indicators alone are insufficient to capture the complexity of leadership development within intellectually demanding practice environments. Leadership enactment involves shifts in identity, relational positioning, emotional labour and ethical decision-making that are best understood through qualitative inquiry. While previous studies have examined leadership behaviours and outcomes, less is known about how intellectual disability practice leaders experience leadership development and interpret its influence on their professional practice. This paper reports the qualitative component of the national programme evaluation, drawing on in-depth interviews with practice leaders who completed the Clinical Leadership Programme. Through inductive qualitative content analysis, the study explores leadership as lived and enacted rather than solely measured, contributing to a deeper understanding of leadership development within intellectual disability services. By foregrounding participants’ experiences, the study extends existing practice leadership literature beyond behavioural measurement to examine leadership as a developmental, relational and ethically situated process. In doing so, it addresses an important gap in the literature and offers implications for leadership education, workforce development and leadership sustainability within intellectual disability services. In response to these gaps, the study was guided by the following research questions: How do practice leaders in intellectual disability services experience participation in a Clinical Leadership Programme? and How do participants perceive the programme as influencing their leadership identity and everyday leadership practice?

## Methods

This paper reports the qualitative component of a national mixed‑methods evaluation of a Clinical Leadership Programme for Persons in Charge working in intellectual disability services in the Republic of Ireland. The overall study design, programme development and quantitative outcomes have been reported previously [13]. The qualitative strand was undertaken to explore participants’ experiences of the programme and to gain an in‑depth understanding of how leadership learning was interpreted, enacted and constrained within practice contexts. A qualitative descriptive design [14] was employed, using individual semi‑structured interviews to capture participants’ perspectives and meanings associated with leadership development. This approach was considered appropriate given the study’s focus on practice‑based experiences and the limited existing qualitative literature exploring practice leadership from the viewpoint of intellectual disability practice leaders.

Ethical approval for the study was granted by the University of Limerick Faculty of Education and Health Sciences Research Ethics Committee (Reference: 2018_10_07_EHS). All procedures adhered to the principles of respect for autonomy, confidentiality, beneficence and non‑maleficence. Participants received written and verbal information outlining the purpose of the study, what participation would involve and their right to withdraw at any time without consequence. Written informed consent was obtained prior to data collection. To protect confidentiality and anonymity, identifying information was removed from transcripts and participants were assigned numerical identifiers. Audio recordings and transcripts were stored securely on password‑protected devices in accordance with institutional data protection policies, with access restricted to the research team.

Participants were drawn from the larger cohort of individuals who completed the Clinical Leadership Programme across four programme deliveries. Eligibility criteria for participation in the qualitative interviews included: (i) completion of the leadership programme, (ii) current employment in an intellectual disability service and (iii) holding a leadership or management role with Person in Charge responsibilities or equivalent. Of the 102 eligible programme graduates, 30 expressed an interest in participating. Fourteen participants subsequently consented and were interviewed between 15 June 2020 and 18 September 2020. Ten interested participants were unavailable when contacted to arrange interviews and six were on leave at the time of recruitment. Participant selection combined purposive sampling and voluntary self-selection to achieve representation across programme cohorts, professional backgrounds, service settings and provider organisations. Participants represented all four programme cohorts (Cohort 1, n = 3; Cohort 2, n = 4; Cohort 3, n = 4; Cohort 4, n = 3) and a range of statutory and voluntary service providers. Participants included intellectual disability nurses, mental health nurses and social care leaders working across residential, community and mixed-service settings. Participants had completed the programme at different time points (December 2018, May 2019, December 2019 and May 2020). As interviews were conducted between June and September 2020, the interval between programme completion and interview ranged from 3 to 18 months. The diversity of professional backgrounds, organisational contexts and programme cohorts supported exploration of both shared and divergent experiences of leadership development.

Data were collected through individual semi-structured interviews conducted either online via secure video-conferencing platforms or face-to-face, depending on participant preference, geographical location and public health restrictions associated with the COVID-19 pandemic. This flexible approach supported accessibility while maintaining consistency in data collection procedures. An interview guide was developed by the research team, informed by the programme learning outcomes and the broader evaluation aims outlined by Doody et al. [13]. Topics explored included experiences of the leadership programme, perceived personal and professional impact, changes in leadership practice, peer and organisational influences and challenges associated with applying learning within practice settings. The semi-structured format ensured consistency across interviews while allowing participants to elaborate on issues they considered most relevant. Interviews lasted between 45 and 75 minutes, were audio-recorded with participants’ consent, transcribed verbatim and checked for accuracy prior to analysis. Interviews were conducted by MoH, an independent research assistant with experience in qualitative research. Although members of the wider research team were involved in the design, delivery and evaluation of the Clinical Leadership Programme, the interviewer was not involved in programme delivery, participant assessment, or line management and held no professional authority over participants. While some participants were known professionally to members of the research team through the national leadership initiative, no direct reporting or supervisory relationships existed. To minimise the potential influence of social desirability bias, participants were informed that honest feedback, including criticism of the programme, was welcomed and that participation would have no impact on their professional roles or engagement with programme stakeholders. Reflexive notes were maintained throughout data collection to document assumptions, interviewer reflections, participant interactions and emerging interpretations.

Data were analysed using inductive qualitative content analysis following the approach described by Elo and Kyngäs [15]. This method was selected to enable themes to emerge from participants’ accounts rather than being imposed a priori. Analysis proceeded through three interconnected phases: preparation, organisation and abstraction. During the preparation phase, transcripts were read repeatedly to facilitate immersion in the data. Meaning units relevant to leadership development and leadership practice were identified and coded. In the organising phase, codes were compared for similarities and differences and grouped into subcategories. These subcategories were subsequently abstracted into higher-order categories and overarching themes reflecting the latent content of the data. Analysis was iterative, involving movement between the raw data, codes, categories and emerging interpretations.

To enhance trustworthiness, coding and theme development were discussed within the research team, analytic decisions were documented to provide an audit trail and verbatim quotations were incorporated to support transparency and demonstrate the relationship between participants’ accounts and the findings. The analysis actively sought disconfirming and divergent cases during coding and theme development. Reflexivity was embedded throughout the analytic process. The research team engaged in ongoing reflexive dialogue, critically examining how prior involvement in leadership development research and familiarity with intellectual disability services might influence data interpretation, coding decisions and theme development. Consistent with principles of qualitative description and qualitative content analysis, emphasis was placed on depth, meaning and contextual understanding rather than frequency of occurrence.

The Clinical Leadership Programme [13] was specifically designed for nurse and social care managers working within intellectual disability services in Ireland. Developed using evidence from the literature, practice experience and national expertise, the programme was accredited for 35 Continuing Education Units (CEUs) and aimed to strengthen participants’ understanding and application of leadership within contemporary intellectual disability services. Delivered over 5.5 days over six months, the programme combined structured teaching, experiential learning, reflective practice, action learning and project work. Learning activities progressed from exploring leadership concepts and self-awareness to applying leadership principles within participants’ own practice settings. The programme culminated in a forum day during which participants presented group projects and shared learning. Key learning outcomes included:

- developing leadership knowledge and understanding within intellectual disability services;
- enhancing communication and interpersonal leadership skills;
- promoting person-centred practice and advocacy;
- strengthening the ability to support, empower and develop staff;
- applying leadership principles to service improvement initiatives; and
- fostering collaborative approaches to team leadership.

A variety of teaching approaches were used, including facilitated workshops, presentations, experiential learning activities, reflective exercises, action learning and group discussion. Participants were encouraged to critically examine their leadership practices, identify development needs and address real-world leadership challenges within their services. Opportunities for peer networking and collaborative learning were embedded throughout the programme, enabling participants to share experiences and develop professional support networks across organisations and service settings. Assessment included engagement in programme activities, completion of individual and group learning tasks and participation in the final forum day.

### Findings

Fourteen participants contributed to the study and Table 1 provides an overview of the participant profile including professional role, background and service context. This contextual information situates the findings and demonstrates the diversity of leadership roles and practice settings represented within the sample. The analysis generated seven themes (Table 2) describing participants’ experiences of leadership development following participation in the Clinical Leadership Programme. While presented separately for reporting purposes, the themes were closely interconnected and collectively reflected the multifaceted nature of leadership development within intellectually demanding, resource-constrained and relationally complex intellectual disability services. Although each theme captures a distinct aspect of participants’ experiences, considerable overlap and interaction were evident across themes. Together, Tables 1 and 2 provide the contextual and analytic foundation for the thematic findings presented below.

**Table 1:** Participant profile.

ID	Sex	Role	Setting	Profession
P1	Female	CNM2 / PIC	Congregated / Community	RNID
P2	Female	CNM2	Adult / Child Respite	RNID
P3	Female	CNM3	Community	RNID
P4	Male	PIC / Manager	Residential	RNID
P5	Female	CNM2	ID / MH Setting	MH Nurse
P6	Female	PIC / Manager	Community / Residential	Social care
P7	Female	Manager	Residential	Social care
P8	Male	CNM2	Congregated	RNID
P9	Female	CNM2	Residential	RNID
P10	Female	CNM2	Day services	MH Nurse
P11	Female	PIC	Residential	Social care
P12	Female	PIC / Manager	Residential	RNID
P13	Female	Manager	Residential	RNID
P14	Female	Manager	Congregated	Social care

* CNM – Clinical Nurse Manager, ID – Intellectual Disability, MH – Mental Health, PIC – Person in Charge, RNID – Registered Nurse Intellectual Disability

**Table 2:** Theme representation and development.

Theme	Generic Category	Sub-Categories	Codes	Meaning Units (examples)
**Transformative personal and professional growth**	Personal transformation	Personal growth	Increased confidence	“It gave me confidence I didn’t know I had”
**Developing reflective and self‑aware leaders**	Self-awareness	Reflective practice	Reflection	“It forced me to stop and think”
**Collective identity and reduced professional isolation**	Collegial support	Peer validation	Shared experience	“We realised we’re all in the same boat”
**Shifting leadership practice towards relational leadership**	Practice change	Leadership skills	Adaptive leadership	“I manage people differently now”
**Extending person‑centredness to staff and teams**	Ethical leadership	Valuing staff	Staff PCC	“We don’t do person centredness for ourselves”
**Systemic pressures constraining leadership practice**	Role tension	System constraints	Burden	“There’s too much paperwork, not enough time”
**Re‑engagement with education and career progression**	Professional development	Lifelong learning	Motivation	“It made me want to go back to college”

To support interpretation of the thematic findings and illustrate relationships identified across participant accounts, Fig 1 presents a conceptual framework grouping the seven themes into four overarching domains. The Fig was developed to aid interpretation of the findings and does not represent a causal, temporal or sequential model. To retain the analytical distinctiveness of each theme while acknowledging areas of conceptual overlap, the themes were organised into four overarching domains (Fig 1).

**Fig 1:**
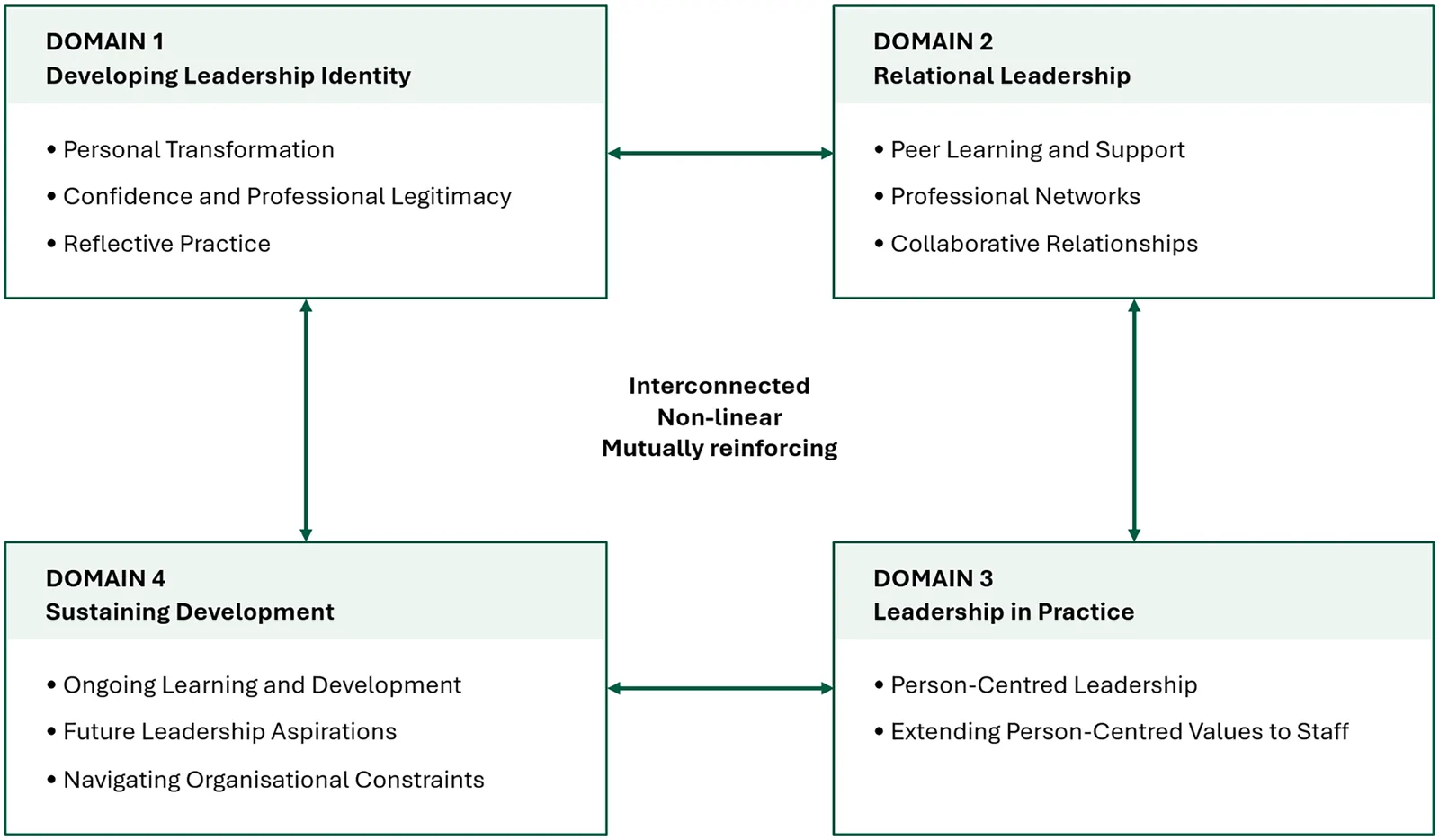
Conceptual framework of leadership development experiences in intellectual disability services.

As illustrated in Fig 1, participants described leadership development as a dynamic and interconnected process. Reflective practice was frequently associated with greater self-awareness, confidence and changes in leadership approach, while peer learning and professional networks provided important sources of validation and support. Participants also described extending person-centred values to staff relationships while navigating organisational and system-level constraints. These interrelationships are explored in greater detail within the thematic findings that follow.

### Transformative personal and professional growth

Participation in the Intellectual Disability Leadership Programme was experienced by participants as transformative rather than incremental. Rather than describing the programme as a means of simply acquiring new knowledge or technical skills, participants articulated a deeper shift involving changes to self‑perception, confidence and professional identity. This transformation was consistently framed as both personal and professional, suggesting that leadership development was experienced as a whole‑person process rather than a discrete role enhancement. Across the data set, participants reflected that they had often assumed leadership roles gradually, typically progressing from clinical or frontline positions into management without structured preparation or formal leadership development. As a result, many entered the programme carrying uncertainty about their legitimacy as leaders and their capacity to meet role expectations. Within this context, the programme functioned as a developmental learning space, enabling participants to integrate extensive experiential knowledge with formal leadership concepts and shared reflection. Several participants described beginning the programme with low self‑confidence, frequently linked to prolonged absence from formal education or entrenched beliefs that leadership capability was innate rather than learned. One participant captured this sense of apprehension clearly:

*“I didn’t have confidence going into it at all… I honestly didn’t think I’d be able for it.” (P1, CNM2/PIC)*

Through structured engagement and interaction with peers who occupied similar leadership positions, participants came to recognise their uncertainty not as personal deficiency but as a common feature of leadership transition within intellectual disability services. This collective experience contributed to a growing sense of reassurance and self‑belief:

*“It reassured me that what I’m doing day to day actually makes sense.” (P4, PIC/Manager)*

Participants described confidence as grounded in self-assurance and professional legitimacy rather than hierarchical authority. Instead, it emerged as a quiet, grounded assurance, enabling leaders to act with greater intentionality, to trust their judgement and to engage more decisively with complex situations. A central element of this transformation was the validation of tacit knowledge. Participants frequently reported that the programme helped them recognise that many of their instinctive practices were underpinned by evidence and established leadership principles, even if they had not previously been articulated as such. As one participant reflected:

*“I realised a lot of what I was doing was evidence‑based, I just didn’t have the language for it before.” (P11, PIC)*

This validation was particularly significant within the intellectual disability service context, where leadership expertise is often undervalued or rendered invisible amid regulatory demands, administrative workload and competing service pressures. For some participants, the absence of feedback or recognition in their roles had contributed to self‑doubt and diminished professional confidence. Participants described the programme as helping to counter this experience by explicitly acknowledging the complexity and significance of leadership work in intellectual disability services:

*“You don’t often get told you’re doing a good job in this area. This made me see that we actually are.” (P9, CNM2)*

Through this process, participants described a shift in how they understood themselves as leaders. What had previously felt like “falling into” leadership roles were reinterpreted as active, competent leadership practice. The programme supported an identity transition from what might be described as an accidental leader to an intentional leader, one who possesses both experiential knowledge and conceptual understanding.

*“I stopped seeing myself as somebody trying to do the role and started seeing myself as a leader.” (P8, CNM2)*

This consolidation of identity appeared to strengthen participants’ sense of professional legitimacy and foster resilience in the face of ongoing role complexity and system pressure. This theme reflects a deep synthesis of personal confidence, professional validation and identity development. The Intellectual Disability Leadership Programme enabled participants not only to recognise their existing capabilities but also to locate themselves more securely within their leadership roles, contributing to participants’ reported confidence, engagement and commitment at the time of interview. While most participants described substantial increases in confidence, some emphasised that confidence remained context dependent. Participants working in highly regulated or resource-constrained environments reported that confidence gained through the programme did not necessarily translate into greater organisational influence.

*“I felt more confident personally but some of the organisational barriers are still there.” (P12, PIC/Manager)*

### Developing reflective and self‑aware leaders

Reflection emerged as a central mechanism through which leadership development occurred, underpinning participants’ reported changes in thinking, behaviour and relational practice. Rather than being described as a peripheral or academic exercise, reflection was experienced as a fundamental leadership capacity that enabled participants to interrogate deeply embedded assumptions, habitual responses and emotional reactions within the complexity of everyday leadership practice in intellectual disability services. Across the data, participants consistently contrasted reflective leadership with what they described as the prevailing culture of constant activity and task completion within intellectual disability services. Leadership roles were commonly portrayed as reactive, characterised by short‑term problem solving and continuous crisis management, leaving little space for thoughtful deliberation or learning:

*“You’re just firefighting the whole time… you don’t get a chance to step back.” (P7, Manager)*

Within this context, reflection had often been perceived as unrealistic, indulgent, or incompatible with operational demands. The programme challenged this view by explicitly positioning reflection as a legitimate and necessary leadership activity, reframing it as integral to effective decision‑making rather than separate from it. Participants described the programme as creating both permission and structure to pause, step back and critically examine their practice:

*“It forced me to stop and think instead of just reacting.” (P12, PIC/Manager)*

This shift was experienced as particularly significant, enabling participants to move away from automatic responses toward more intentional and considered leadership actions. A key aspect of reflective development involved heightened self‑awareness, particularly in relation to emotional responses to staff issues, conflict and organisational pressure. Through guided reflection, participants became increasingly attuned to how their own emotions and stress responses influenced interactions with staff and shaped leadership outcomes:

*“I started noticing my own triggers and how that affected how I spoke to staff.” (P6, PIC/Manager)*

This awareness marked a departure from previously unexamined patterns of behaviour, allowing participants to recognise the interpersonal impact of their leadership style. In turn, this increased self‑awareness translated into tangible changes in practice, including more measured communication, improved patience and greater emotional regulation:

*“I’m calmer now. I don’t escalate things without meaning to.” (P3, CNM3)*

Participants described these changes not as isolated skills but as part of a broader capacity to lead relationally and ethically under pressure. Importantly, reflection was not confined to individual practice. Several participants reported consciously embedding reflective processes within their teams, signalling a shift from task‑focused management toward collective sense‑making and shared learning. Rather than limiting team engagement to operational matters, leaders began facilitating reflective dialogue within routine structures:

*“We now reflect together at team meetings instead of just listing tasks.” (P14, Manager)*

Through these practices, reflection evolved into a collective learning process, fostering open communication, mutual understanding and shared responsibility for practice development. Participants perceived this shift as strengthening team cohesion and enhancing problem‑solving capacity within increasingly complex service environments. Reflection appeared to function as a useful leadership skill, enabling participants to draw on emotional awareness, ethical judgement and relational insight in their practice. By legitimising reflection and embedding it within both individual and team contexts, participants described the programme as facilitating a transition from reactive management to reflective leadership, supporting more sustainable and intentional engagement with the demands of intellectual disability services.

*“I’d ask the team now why we did something rather than just telling them what to do.” (P9, CNM2)*

### Collective identity and reduced professional isolation

Participants consistently described leadership roles within intellectual disability services as inherently isolating, particularly for those occupying positions such as Person in Charge and middle management. This isolation was attributed to the liminal nature of leadership roles situated between frontline staff, senior management and regulatory bodies combined with high levels of responsibility and limited opportunities for peer connection. Within this context, the Intellectual Disability Leadership Programme was experienced as creating a shared professional identity, significantly reducing feelings of invisibility, isolation and emotional burden. A recurring feature of participants’ narratives was the relief associated with recognising that many of the challenges they faced were systemic rather than personal failings. Prior to participating in the programme, several individuals reported internalising difficulties related to workload, staffing, or regulatory pressure as indicators of personal inadequacy. Engaging with peers who articulated similar experiences prompted a reframing of these challenges:

*“I genuinely thought I was the problem until I heard others saying the same things.” (P2, CNM2)*

This shared recognition functioned as a powerful form of validation, allowing participants to externalise structural constraints and to contextualise their experiences within broader organisational realities. Rather than diminishing responsibility, this reframing supported participants to engage more critically and compassionately with their roles. Central to the development of collective identity was participants’ experience of the programme as a psychologically safe and non‑judgemental space. Unlike the work environment, the programme environment enabled honest discussion of uncertainty, vulnerability and failure without fear of repercussion:

*“You could say things there you wouldn’t say in your own organisation.” (P8, CNM2)*

This sense of safety supported deeper engagement with learning, as participants felt permitted to question established practices, share doubts and reflect openly on leadership challenges. The ability to articulate these experiences alongside peers fostered trust, mutual understanding and a sense of solidarity rooted in shared professional struggle. Importantly, the sense of connection established during the programme extended beyond its formal boundaries. Many participants described continued engagement through informal peer networks, most commonly sustained via digital platforms:

*“The WhatsApp group never stopped.” (P10, CNM2Manager)*

These ongoing connections functioned as communities of practice, providing spaces for shared problem‑solving, advice‑seeking, emotional support and celebration of successes. Participants described these networks as particularly valuable in moments of uncertainty or isolation within their roles, offering affirmation and perspective that were often unavailable within their own organisations. Through these interactions, participants described the programme as facilitating a shift from individualised leadership experiences toward a collective professional identity, characterised by mutual recognition and shared learning. This collective identity appeared to mitigate emotional labour by distributing it across a peer group rather than leaving individuals to manage burden in isolation. This highlights the relational and emotional dimensions of leadership in intellectual disability services, emphasising that leadership sustainability extends beyond individual competence to include connection, belonging and peer support. The findings suggest that opportunities for shared reflection and professional connection are not merely beneficial adjuncts to leadership development but are foundational to wellbeing, resilience and sustained engagement in complex care environments. Not all participants maintained equally active peer connections following programme completion. While some described ongoing engagement through informal networks, others reported that workload and competing demands limited continued participation.

*“We still had the group, but to be honest once work got busy I wasn’t as involved as I would have liked to be it was difficult to keep in touch regularly because everyone was dealing with their own pressures and workloads.” (P6, PIC/Manager)*

### Shifting leadership practice towards relational leadership

Participants described a fundamental shift in how they enacted leadership, characterised by movement away from directive, task‑oriented approaches toward more relational, adaptive and inclusive leadership practices. This shift was not experienced as an abandonment of authority or accountability; rather, it reflected a reorientation in how leadership was exercised within the relationally complex context of intellectual disability services. Prior to undertaking the programme, several participants described leadership as largely transactional, focused on task completion, compliance and operational oversight. While this approach was acknowledged as necessary in high‑demand service environments, it was frequently associated with tension, resistance and emotional distance between leaders and staff. Through engagement with the programme, participants began to reconceptualise leadership as a relational practice, grounded in understanding, responsiveness and collaboration. A key aspect of this reframing involved recognising staff members as individuals rather than a homogeneous workforce. Participants reported heightened awareness of differences in staff needs, motivations, communication styles and capacities. This awareness led to more nuanced and personalised leadership approaches, replacing uniform management strategies with adaptive relational engagement:

*“I don’t manage the team as a block anymore. I manage people.” (P13, Manager)*

This individualised approach was perceived to enhance trust, engagement and morale, while also reducing misunderstandings and conflict within teams. Conflict itself was subject to significant reframing. Previously experienced as threatening, emotionally charged, or indicative of failure, conflict was reconceptualised through programme learning as a source of information about team dynamics, communication breakdowns and unmet needs. One participant articulated this shift clearly:

*“I learned conflict isn’t personal it’s information.” (P5, CNM2)*

This reframing enabled participants to approach conflict proactively and constructively, addressing issues directly while preserving relationships. Rather than avoiding disagreement or responding defensively, leaders described greater confidence in engaging with difficult conversations in ways that balanced clarity with respect. Participants also reported increased commitment to collaborative decision‑making, viewing staff involvement not as a risk to authority but as a resource for shared ownership and improved outcomes. This marked a departure from earlier practices where change was often implemented through top‑down directives, sometimes leading to resistance or disengagement:

*“I bring staff with me now instead of dragging them.” (P10, CMM2)*

By involving staff in decision‑making processes, practice leaders perceived greater buy‑in, accountability and collective responsibility for practice development. This approach was particularly valued in intellectual disability services, where staff continuity, relationships and experiential knowledge are central to quality care. The enactment of leadership shifted from control‑based management toward relational influence, underpinned by trust, dialogue and mutual respect. Participants described themselves as facilitators of practice rather than sole drivers of change, recognising leadership as distributed across teams rather than residing exclusively within formal roles. This reflects a move toward transformational and distributed leadership orientations, which participants found to be particularly well suited to the emotional, ethical and relational complexities of intellectual disability service environments. The shift toward relational leadership enhanced leaders’ capacity to navigate uncertainty, foster collaboration and sustain engagement within teams facing ongoing systemic pressure. Several participants noted that adopting a more relational approach required additional time and effort and was not always readily accepted within existing organisational cultures.

### Extending person‑centredness to staff and teams

Participants described a conceptual and ethical shift in how they understood person‑centredness, moving beyond its traditional application to service users to recognise that staff also require care, consideration and empowerment. This shift represented a deepening of leadership values, whereby participants began to question long‑standing assumptions about whose wellbeing and autonomy were prioritised within intellectual disability services. Across the data set, participants acknowledged a tension between espoused values and enacted practice. While person‑centredness, empowerment and advocacy were described as foundational principles underpinning care delivery for individuals receiving support from the service, these same principles were not always extended to staff teams. Several participants reflected critically on this dissonance, recognising that staff wellbeing and inclusion had often been secondary to operational demands:

*“We talk about empowerment for clients, but we don’t do it for staff.” (P2, CNM2)*

This realisation emerged as a pivotal moment in participants’ leadership development, prompting reflection on the ethical consistency of their practice. Rather than framing staff‑related challenges solely in terms of performance or compliance, participants began to recognise staff as moral stakeholders within the service, individuals whose dignity, voice and autonomy mattered not only instrumentally but intrinsically. As a result, participants reported reframing their relationship with staff from one oriented toward resource management to one grounded in partnership and mutual respect. Leaders began to view staff not as interchangeable units of labour but as relational actors whose engagement and commitment were shaped by how valued and understood they felt:

*“Staff do better when they feel seen.” (P6, PIC/Manager)*

Participants reported that this reframing contributed to deliberate changes in leadership practice, including increased listening, recognition of effort and inclusion of staff perspectives in decision‑making processes. Participants described becoming more intentional in acknowledging staff contributions, understanding individual circumstances and supporting professional growth. These actions were perceived not as additional burdens but as essential expressions of ethical leadership. Participants consistently linked this shift in orientation to observable changes within their teams. Several described improvements in morale, confidence and initiative, suggesting that extending person‑centred principles to staff fostered a sense of ownership and shared responsibility for practice:

*“People started taking ownership when they felt trusted …* p*eople were speaking up more and bringing ideas forward.” (P3, CNM3)*

Rather than diminishing leadership authority, this approach appeared to strengthen it, as trust and collaboration replaced compliance‑driven engagement. Staff were described as more willing to contribute ideas, engage with change and assume responsibility for service development when they felt respected and empowered. Importantly, this extension of person‑centredness was not described as separate from service‑user outcomes. Participants viewed staff wellbeing and empowerment as contributory to quality care, recognising that relationally attuned leadership created conditions in which staff were better able to practice ethically, compassionately and consistently with service‑user values. This theme reflects a process of ethical leadership alignment, in which participants sought coherence between the values they promoted for service users and the values enacted within their teams. By extending principles of dignity, respect, participation and empowerment throughout the organisation, participants moved toward a more holistic and sustainable model of leadership suited to the relational and ethical complexity *or ‘modern day principles’* of intellectual disability services.

### Systemic pressures constraining leadership practice

Despite the substantial personal and professional growth reported by participants, there was a consistent and compelling recognition that the application of leadership learning was constrained by systemic and organisational pressures. Participants articulated a clear awareness that while the Intellectual Disability Leadership Programme enhanced their capacity to lead reflectively, relationally and ethically, these capacities were often enacted within systems that were not fully aligned with such approaches. This resulted in a persistent tension between leadership ideals and operational realities. Administrative burden was the most frequently cited constraint. Participants described documentation requirements as increasingly dominant in their roles, often displacing time allocated to staff engagement, reflection and direct leadership practice:

*“Paperwork has taken over everything.” (P7, Manager)*

The volume and complexity of documentation, largely driven by regulatory and governance demands, were perceived as both time‑consuming and emotionally draining. Leaders expressed frustration that the work most visible within systems was often administrative compliance rather than relational or practice leadership, despite the latter being central to service quality. Participants also described significant role overload and an incremental expansion of responsibilities, reporting that leadership positions frequently involved absorbing responsibilities beyond their formal remit. Tasks traditionally associated with other professional groups or organisational levels were often redirected to frontline leaders, limiting capacity to focus on strategic leadership functions:

*“You end up doing everyone else’s job.” (P8, CNM2)*

This accumulation of responsibilities contributed to fatigue, role strain and a sense of being perpetually reactive rather than proactive. For some participants, this undermined their ability to sustain reflective practices introduced during the programme, reinforcing cycles of task‑driven leadership. A further layer of constraint emerged through participants’ experiences of values misalignment between leadership ideals and system priorities. While leaders described increased commitment to person‑centred, staff‑inclusive and relational leadership, they often perceived organisational systems as prioritising efficiency, compliance and risk management over relational and ethical considerations:

*“You want to be person centred, but the system isn’t.” (P9, CNM2)*

This misalignment generated professional tension, as leaders attempted to reconcile deeply held values with externally imposed expectations. Participants described instances where person‑centred initiatives or staff‑focused leadership practices were difficult to sustain due to staffing shortages, resource constraints, or performance metrics that failed to capture relational quality. Importantly, participants did not frame these constraints as insurmountable failings of leadership development. Rather, they demonstrated sophisticated insight into the interaction between individual agency and structural limitation. The challenge was not a lack of leadership competence, but the absence of complementary organisational conditions necessary to support and sustain leadership practice. This highlights a leadership–system mismatch, suggesting that leadership development initiatives, while impactful at the individual level, must be accompanied by organisational alignment to achieve sustained change. Participants’ accounts suggest that without structural supports such as workload redistribution, protected reflective time and value‑consistent governance leaders risk experiencing frustration, erosion of gains, or burnout over time. Participants’ experiences highlight the necessity of viewing leadership not solely as an individual capability but as a system‑enabled practice, requiring congruence between leadership development, organisational culture and structural design within intellectual disability services.

### Re‑engagement with education and career progression

Participation in the Intellectual Disability Leadership Programme prompted a marked re‑engagement with education and career development, particularly among participants who had been long removed from formal academic study. This re‑engagement was not described merely as renewed interest in education for its own sake, but as a significant shift in how participants understood learning as integral to leadership capability, confidence and sustainability within intellectually demanding service environments. Many participants reflected on prior disengagement from education, often rooted in negative past experiences, fear of academic assessment, or perceptions that further study was incompatible with professional and personal responsibilities. For some, these fears had become entrenched over time, reinforcing the belief that advanced education was inaccessible or unnecessary at later stages of their careers:

*“I was terrified of college before this.” (P11, PIC)*

The programme challenged these beliefs by providing a supportive, practice‑focused learning environment that was clearly aligned with participants lived leadership experiences. Rather than positioning education as abstract or detached from practice, the programme emphasised relevance, application and collective learning. This approach reduced anxiety and fostered confidence, enabling participants to reconceptualise themselves as capable learners. As confidence grew, participants began to articulate a renewed desire for continued learning and progression, often describing the programme as a catalyst rather than an endpoint:

*“I want to keep going now.” (P14, Manager)*

Importantly, this shift was not framed as individual ambition divorced from organisational or service needs. Instead, participants increasingly came to view education as a strategic leadership investment, recognising that ongoing development was essential for sustaining effective leadership in the face of evolving regulatory, professional and service demands. Education was perceived as strengthening decision‑making capacity, resilience and professional legitimacy. Participants also described how engagement with the programme reframed their understanding of career trajectories within intellectual disability services. Leadership development was no longer conceptualised as a static endpoint associated with managerial appointment, but as a dynamic, evolving process requiring continuous learning.

*“I never saw leadership as something you kept developing. I thought once you got the post that was it. Now I realise you’re always learning.” (P4, PIC/Manager)*

This reframing appeared particularly significant in a sector where opportunities for structured leadership development have historically been limited. Beyond individual benefit, participants recognised the broader implications of re‑engagement with education for the sustainability of intellectual disability services. Renewed interest in further study was linked to aspirations for improved leadership succession, enhanced practice quality and the future capacity of the workforce. Participants expressed a heightened sense of responsibility not only to their own professional growth but to modelling lifelong learning for colleagues and emerging leaders.

*“It opened my eyes to opportunities I hadn’t considered before.” (P13, Manager)*

This theme represents an important dimension of leadership sustainability, highlighting how accessible, relevant leadership programmes can reawaken educational identity and motivate continued professional development.

*“I never thought I’d go back learning again but it reminded me that I actually enjoy it.” (P3, CNM3)*

By legitimising learning as an essential component of leadership, participants perceived the programme as contributing to future leadership capacity within intellectual disability services and reinforcing the long‑term viability of leadership roles characterised by complexity, emotional labour and systemic constraint. Although participants broadly described positive experiences of the programme, variation was evident in the extent to which learning could be enacted in practice. Participants working within highly resource-constrained environments reported greater difficulty translating learning into leadership practice, particularly where workload pressures, staffing shortages or organisational priorities limited opportunities for reflection and staff engagement. While no participant viewed the programme as ineffective, several described frustrations regarding organisational barriers that constrained implementation. These variations informed theme development and were considered alongside convergent accounts during analysis.

## Discussion

This qualitative study extends the mixed-methods evaluation reported by Doody et al. [13] by examining how practice leaders experienced and interpreted participation in a Clinical Leadership Programme. While the quantitative findings demonstrated improvements in leadership confidence, perceived competence and career-related outcomes, the present study provides insight into the processes participants associated with these changes. Participants described leadership development as a relational, reflective and identity-based experience situated within the realities of contemporary intellectual disability services. This shift towards a more secure leadership identity aligns with contemporary leadership scholarship that conceptualises leadership as an evolving sense of self, shaped through experience, reflection and relational engagement rather than through technical competence alone [10,16]. Many participants had entered leadership roles incrementally, often without formal preparation, reflecting wider patterns identified within intellectual disability services where leadership responsibility frequently emerges through clinical progression rather than structured development pathways [3,17]. Within this context, participants described the programme as providing a legitimising space in which participants could reconcile experiential knowledge with formal leadership language, thereby strengthening confidence, professional identity and resilience.

The recognition and validation of tacit knowledge was particularly significant. Participants described gaining a language to articulate leadership practices they were already enacting but had not previously conceptualised as leadership. This finding resonates with Manley et al.’s [9] assertion that practice leadership is grounded in experiential, moral and relational knowledge embedded within everyday work. Such validation is especially important within intellectual disability nursing, a profession that has historically struggled with visibility, status and recognition within broader healthcare systems [11,18]. The consolidation of leadership identity evident in this study may therefore represent a critical mechanism for sustaining leadership capacity within a profession characterised by high emotional labour and systemic pressure. Reflection emerged as a core mechanism underpinning leadership development. Participants described a shift away from reactive, task‑driven leadership towards more intentional, reflective practice, enabling greater awareness of emotional responses, communication patterns and decision‑making behaviours. This aligns with long‑standing assertions that reflective practice is foundational to effective leadership in complex, emotionally demanding care settings [10]. Participants described reflection as supporting individual self‑awareness but also relational leadership capacity, supporting improved communication, emotional regulation and ethical judgement, consistent with earlier work linking reflective leadership to compassionate and values‑driven care [11].

Importantly, participants described the programme as legitimising reflection as a leadership responsibility rather than a discretionary activity, challenging the pervasive culture of constant crisis management and task saturation reported in intellectual disability services [1,2]. Participants’ accounts suggest that when reflection is structured, valued and supported, it becomes a conduit for more deliberate leadership enactment. The extension of reflective practices into team contexts further supports distributed leadership models, reinforcing collective learning and shared responsibility for practice quality [19]. Participants’ experiences also highlight the isolating nature of leadership within intellectual disability services, particularly for those in middle‑management positions navigating competing organisational, regulatory and staff demands. Recognition that such challenges were shared rather than personal provided significant emotional relief and reframed perceived inadequacies as systemic issues, echoing previous research identifying structural rather than individual origins of leadership strain in disability services [3,20]. The psychologically safe learning environment fostered during the programme enabled openness, vulnerability and mutual validation, facilitating peer connections that extended beyond programme completion and functioned as informal communities of practice. This finding extends the predominantly staff‑focused practice leadership literature by foregrounding the relational needs of leaders themselves. Peer connection appears to be a critical yet under‑recognised resource for leadership sustainability, especially within emotionally demanding and highly regulated contexts [7,8]. Such networks were described as important sources of support, affirmation and shared problem-solving and may contribute to sustaining leadership engagement in demanding practice environments.

Leadership enactment following programme participation was characterised by a clear shift towards relational and inclusive practices. Participants described reframing leadership from directing work to facilitating engagement, recognising staff as individuals with distinct needs, strengths and communication styles. This relational orientation aligns with distributed and transformational leadership theories increasingly advocated within healthcare [21,22] and is consistent with practice leadership literature emphasising modelling, coaching, feedback and presence as mechanisms for improving staff practice and service user outcomes [5,6]. Together, these findings suggest that leadership development was not experienced as a shift in authority but as a shift in how authority was exercised. Participants appeared to move from viewing leadership primarily as oversight and control towards understanding leadership as a relational process of influence, engagement and partnership. The reframing of conflict as a source of information rather than threat is particularly notable, given the frequency with which intellectual disability leaders manage emotionally charged situations involving staff stress, behaviours of concern and regulatory scrutiny [23]. Ethical leadership emerged as a central consideration, particularly through participants’ recognition of a dissonance between advocating person‑centredness for service users and insufficiently extending the same values to staff. Reframing staff as partners rather than resources reflects ethical and relational leadership frameworks and reinforces evidence linking staff empowerment with quality care delivery [9,24]. This finding highlights participants’ perceptions of an important relationship between staff wellbeing, staff engagement and the delivery of person-centred support, strengthening arguments that person‑centred care must be enacted consistently across organisational relationships [5,7].

Despite these positive developments, participants described significant systemic constraints limiting the enactment of leadership learning in practice. Administrative burden, role overload and compliance‑driven organisational cultures were seen to conflict with relational and reflective leadership practices. These findings echo longstanding concerns regarding leadership–system misalignment within intellectual disability services, where regulatory and governance demands may inadvertently displace relational leadership despite policy commitments to person‑centred care [3,20]. Importantly, participants did not frame these tensions as individual leadership failures but as structural limitations, reinforcing calls for organisational alignment if leadership development is to achieve sustained impact [19,25]. Finally, re‑engagement with education emerged as a marker of leadership sustainability. For many participants, the programme reactivated an educational identity that had been dormant, reframing learning as a strategic investment in leadership capacity rather than a personal or academic burden. This aligns with evidence that accessible, practice‑relevant education strengthens leadership resilience, succession planning and professional credibility within under‑resourced sectors [26,27]. Such re‑engagement has broader implications for the future of intellectual disability staff, supporting the development of research‑informed leaders and enhancing the profession’s visibility and influence. Taken together, these findings advance understanding of practice leadership in intellectual disability services by foregrounding leadership as an identity‑based, relational and contextually constrained process. The study complements the quantitative outcomes reported by Doody et al [13]. by elucidating the mechanisms through which leadership development is internalised and enacted. By centring leaders lived experiences, this paper addresses a critical gap in the literature and provides empirical support for leadership development initiatives that are relationally grounded, ethically aligned and supported by organisational systems.

Beyond demonstrating perceived benefits of leadership education, this study contributes to leadership development theory by illustrating how leadership identity is shaped through reflection, peer validation, relational learning and organisational context. In doing so, it extends existing intellectual disability leadership literature, which has largely focused on leadership behaviours and outcomes, by examining the developmental processes through which leaders come to understand and enact leadership. Unlike leadership development research that emphasises individual competency acquisition, participants placed greater emphasis on identity formation, peer connection and reflective practice as key mechanisms of development. As illustrated in Fig 1, leadership development was described as an interconnected rather than linear process, encompassing identity formation, relational engagement, leadership enactment and ongoing professional development. Although participants’ accounts were largely convergent, variation existed in the extent to which learning could be enacted within practice settings, with those working in more resource-constrained environments describing greater challenges in translating learning into practice. Together, these findings advance understanding of leadership development within intellectual disability services as an identity-based, relational and contextually constrained process and highlight the importance of organisational systems in supporting the enactment of leadership learning.

### Implications

Implications for practice, the findings suggest that practice leadership in intellectual disability services may be strengthened when enacted as a relational, reflective and ethically grounded practice, rather than as a purely managerial or compliance‑driven function. Participants described enhanced confidence, visibility and legitimacy when they were supported to engage reflectively with their role and to lead with staff rather than over them. Practice environments should therefore enable leaders to prioritise relational work, including staff engagement, supervision, reflection and modelling of person‑centred values. Organisations should recognise that leadership presence at the point of practice is central to quality care. Strategies such as protected time for leadership activities, reduced administrative burden where possible and meaningful team reflection forums may strengthen leaders’ capacity to sustain relational leadership. Additionally, extending person‑centred principles to staff as moral stakeholders rather than functional resources has implications for workforce morale, retention and care quality, reinforcing leadership as an ethical as well as operational responsibility. Peer connection emerged as a critical mechanism for leadership sustainability. Services should actively support peer networks, mentoring arrangements and communities of practice for intellectual disability practice leaders to mitigate professional isolation and promote shared learning.

Implications for education, leadership education for intellectual disability staff should move beyond generic or competency‑based programme models and instead adopt context‑specific, practice‑based and reflective approaches. The findings suggest that leadership learning is most meaningful when participants can integrate theory with lived experience, share practice narratives and legitimise tacit knowledge developed over years of practice.

Educational provision should explicitly address leadership identity, emotional labour, relational competence and ethical decision-making while creating opportunities for participants to integrate theory with lived experience and legitimise tacit leadership knowledge developed through practice. Importantly, leadership programmes can act as a gateway for re‑engagement with formal education among experienced practitioners, reframing learning as an investment in leadership sustainability rather than an additional burden. Partnerships between higher‑education institutions, service providers and clinical leadership bodies are essential to ensure that leadership education remains accessible, accredited and relevant to contemporary service realities. Embedding qualitative reflection, peer dialogue and work‑based initiatives should be prioritised in future programme design.

Implications for policy, the findings highlight a persistent leadership–system misalignment, where leadership development is encouraged at an individual level but constrained by organisational and regulatory structures. Policymakers should recognise that leadership capacity may be difficult to sustain without parallel investment in system‑level supports. Leadership development initiatives should be accompanied by policies that recognise supervision, staff development and reflective practice as legitimate leadership activities. Without alignment between leadership development programmes and organisational structures, the potential benefits of leadership learning may be difficult to sustain in practice. Regulatory frameworks should seek to balance accountability and quality assurance with flexibility that allows leaders to prioritise person‑centred and staff‑inclusive practices. Without such alignment, leadership development risks becoming performative rather than transformative. Furthermore, recognising and strengthening leadership pathways for intellectual disability staff is essential for succession planning, workforce retention and service quality within a sector facing increasing complexity and demand.

Implications for research, this study demonstrates the value of qualitative inquiry in advancing understanding of practice leadership in intellectual disability services. Future research should build on these findings through longitudinal designs examining how leadership development is maintained over time and how participants perceive its influence on organisational culture, staff experiences and service delivery. Inclusion of multiple stakeholder perspectives including frontline staff, service users and families would enhance understanding of how leadership development is experienced relationally and systemically. Additionally, comparative studies across service models and jurisdictions would support examination of how contextual factors shape leadership enactment. Finally, further research is warranted to explore how leadership development programmes can be integrated with organisational change initiatives, ensuring that leadership learning is both supported and embedded within practice systems.

### Limitations

Several limitations should be considered when interpreting the findings of this study. First, the qualitative sample comprised a relatively small group of participants who had completed the Clinical Leadership Programme and voluntarily agreed to participate in follow-up interviews. While purposive sampling supported depth and richness of data, participants’ accounts may reflect more positive or engaged perspectives on leadership development. Individuals who experienced the programme less favourably or who encountered greater difficulties applying learning within practice may be under-represented. Second, researcher positionality warrants consideration. Members of the research team were involved in programme development and evaluation and although reflexive discussions, an audit trail and collaborative coding were used to challenge assumptions and support confirmability, familiarity with the programme may have influenced interpretation of participant accounts and theme development. Third, data were generated through self-reported interviews and therefore reflect participants’ perceptions rather than direct observations of leadership practice. Participant accounts may have been influenced by recall bias or social desirability, particularly given the positive nature of the programme experience. While efforts were made to create a non-judgemental interview environment and encourage critical reflection, participants may have emphasised positive changes or attributed developments to the programme.

Fourth, participants were interviewed at varying intervals following programme completion, ranging from approximately 3–17 months. Consequently, participant recall and reflections may have differed according to the time elapsed since programme completion. The study therefore captures participants’ perceptions of leadership development at a single point in time rather than longer-term leadership enactment or sustained organisational change. Longitudinal qualitative research would provide greater insight into how leadership learning is maintained, adapted or challenged over time. Fifth, data collection occurred during the COVID-19 pandemic, a period characterised by heightened service pressures, workforce strain, altered working arrangements and increased professional isolation. These contextual factors may have influenced participants’ leadership experiences, reflections on programme impact and opportunities to apply learning within practice. Finally, the study was conducted within the specific regulatory, organisational and policy context of intellectual disability services in the Republic of Ireland. Leadership experiences are influenced by national governance structures, workforce arrangements and service models and transferability to other jurisdictions should therefore be considered cautiously. In addition, the study captures the perspectives of practice leaders only. Inclusion of staff, service users and family members would strengthen understanding of how leadership development is experienced across organisational and care relationships and how participants’ perceptions align with the experiences of others.

## Conclusion

This study contributes to understanding leadership development within intellectual disability services by foregrounding the experiences of practice leaders and examining leadership as an identity-based, relational and contextually situated process. Extending previous quantitative findings [13], the study provides insight into how participants understood and experienced leadership development following participation in a national Clinical Leadership Programme. Rather than describing leadership development solely in terms of skill acquisition, participants associated leadership growth with increased confidence, professional legitimacy, reflective practice and relational engagement. Peer connection was viewed as an important source of support, while ongoing organisational pressures highlighted the challenges of enacting leadership learning within complex service environments. The findings suggest that leadership development in intellectual disability services is not reducible to the acquisition of technical competencies. Rather, it involves the consolidation of leadership identity, the validation of tacit and experiential knowledge and the cultivation of reflective and relational capacities that enable leaders to navigate complexity, ethical tension and emotional labour. Reflection emerged as a critical enabler of intentional leadership practice, while peer connection mitigated professional isolation and supported leadership sustainability. At the same time, the study highlights the enduring impact of systemic constraints including administrative burden, role overload and compliance‑driven organisational cultures which can undermine the application of leadership learning despite individual growth. By centring the perspectives of practice leaders, this study extends existing practice leadership literature beyond behavioural descriptions of leadership and contributes to understanding how leadership identity is developed and enacted within intellectual disability services. Future research should examine how these experiences evolve over time and explore the perspectives of staff, service users and families to further understand the wider influence of leadership development within practice settings.

### AI Use Statement

Artificial intelligence tool (Copilot) was used to support manuscript preparation (editing). The authors reviewed and revised all content to ensure accuracy, integrity and alignment with the intended meaning. The authors take full responsibility for the final content of the manuscript.
