# Supplementary material for: Practice leadership development in intellectual disability services: A qualitative study of practice leaders’ experiences
Source: PLoS One. 2026 Nov 3;21(11):e0351893. doi: 10.1371/journal.pone.0351893 (PMC13730323; doi:10.1371/journal.pone.0351893)
Supplement: Supplementary file 1 [file pone.0351893.s002.docx]

**Response to Editor and Reviewers**

**Manuscript:** Practice Leadership Development in Intellectual Disability Services: A Qualitative Study of Practice Leaders' Experiences

We sincerely thank the Editor and Reviewers for their thoughtful and constructive feedback. We have carefully considered all comments and have revised the manuscript. Revisions have strengthened methodological transparency, enhanced reflexivity, clarified the study's relationship to the previous evaluation, reduced causal language, strengthened evidentiary support through additional quotations, expanded discussion of variation in participants' accounts, and improved the positioning of the findings within the wider leadership development literature. All changes are identified in the revised manuscript using tracked changes (red font).

**Editor Comments**

**Comment 1. Researcher positionality and reflexivity**

**Response**

We thank the Editor for highlighting the importance of reflexivity and positionality. We have substantially expanded the Data Collection, Data Analysis and Limitations sections to provide greater transparency regarding the relationship between the research team, the programme and participants. Specifically, we now identify who conducted the interviews, clarify that the interviewer was not involved in programme delivery, participant assessment or line management, describe any prior professional relationships, explain measures taken to minimise social desirability bias and detail the reflexive processes used throughout analysis. We have also expanded the Limitations section to explicitly discuss how researcher positionality may have influenced coding, interpretation and theme development.

**Changes made**

**Methods: Data Collection**

Added:

Interviews were conducted by MoH, an independent research assistant with experience in qualitative research. Although members of the wider research team were involved in the design, delivery and evaluation of the Clinical Leadership Programme, the interviewer was not involved in programme delivery, participant assessment and held no professional authority over participants. While some participants were known professionally to members of the research team through the national leadership initiative, no direct reporting or supervisory relationships existed.

Added:

Participants were informed that honest feedback, including criticism of the programme, was welcomed and that participation would have no impact on their professional roles or engagement with programme stakeholders.

**Methods: Data Analysis**

Added:

The research team engaged in ongoing reflexive dialogue, critically examining how prior involvement in leadership development research, programme evaluation and familiarity with intellectual disability services might influence data interpretation, coding decisions and theme development.

**Limitations**

Added:

Researcher positionality warrants consideration. Members of the research team were involved in programme development and evaluation and although reflexive discussions, an audit trail and collaborative coding were used to challenge assumptions and support confirmability, familiarity with the programme may have influenced interpretation of participant accounts and theme development.

**Comment 2. Causal and temporal claims**

**Response**

We agree and have revised the manuscript throughout to ensure that findings are presented as participants' perceptions and experiences rather than objective outcomes attributable to the programme. We have replaced causal language with wording such as "participants reported", "participants perceived" and "participants described". We have also removed or revised wording implying longitudinal evidence.

**Changes made**

Examples include:

Original: The programme strengthened staff engagement.

Revised: Participants described greater staff engagement following participation in the programme.

Original: The programme facilitated a transition from reactive management to reflective leadership.

Revised: Participants described a transition from reactive management to reflective leadership.

Original conclusion: By examining how intellectual disability nurse leaders experienced and enacted a national Clinical Leadership Programme, the study moves beyond outcome measurement to reveal the mechanisms through which leadership capacity is formed, sustained, and challenged...

Revised: By examining how practice leaders in intellectual disability services experienced a national Clinical Leadership Programme, the study provides insight into the processes participants associated with the development and enactment of leadership capacity within practice.

**Comment 3. Interpretive claims and supporting evidence**

**Response**

We agree and have strengthened evidentiary support throughout the Results section. Additional participant quotations were incorporated to support key interpretive claims, particularly those relating to educational re-engagement, career progression and leadership identity formation.

**Changes made**

Added quotations including:

"I never saw leadership as something you kept developing. I thought once you got the post that was it. Now I realise you're always learning." (P4)

and

"It opened my eyes to opportunities I hadn't considered before." (P13)

Interpretive statements were also revised where direct evidence was limited.

**Comment 4. Analytic variation**

**Response**

We thank the Editor and Reviewer 1 for raising this important point. We revisited the dataset to identify variation and divergence within participant accounts. While the overall pattern of findings demonstrated considerable convergence, variation was evident in participants' ability to enact leadership learning within different organisational contexts. We also clarified how disconfirming cases were actively sought during analysis.

**Changes made**

**Methods: Data Analysis**

Added:

The analysis actively sought disconfirming and divergent cases during coding and theme development.

**Findings**

Added:

Although participants' accounts were largely convergent, variation existed in the extent to which leadership learning could be enacted within practice settings. Participants working within more resource-constrained environments described greater challenges in translating programme learning into everyday leadership practice.

**Discussion**

Added discussion regarding contextual variation and organisational constraints.

**Comment 5. Data Availability Statement**

**Response**

We have revised the Data Availability Statement to comply with PLOS policy.

**Changes made**

Revised statement:

The qualitative interview dataset contains potentially identifiable information from a small sample of participants involved in a national leadership programme and therefore cannot be publicly shared. Data are held by the University of Limerick in accordance with ethical approval requirements. Requests for access to de-identified data may be directed to the Research Governance Office, University of Limerick, subject to ethical and data governance approval.

**Comment 6. Recruitment and sampling**

**Response**

Additional detail has been provided regarding eligibility, recruitment, sampling and sample adequacy.

**Changes made**

Added:

Of the 102 eligible programme graduates, 30 expressed interest in participating. Fourteen participants subsequently consented and were interviewed. Ten interested participants were unavailable when contacted to arrange interviews and six were on leave at the time of recruitment.

Added:

Participant selection combined purposive sampling and voluntary self-selection.

Added:

Participants represented all four programme cohorts (Cohort 1 n=3; Cohort 2 n=4; Cohort 3 n=4; Cohort 4 n=3) and a range of statutory and voluntary provider organisations.

**Comment 7. Description of the programme**

**Response**

A new programme description paragraph has been included.

**Changes made**

**Clinical Leadership Programme**

Including:

- duration (5.5 days / 35 CEUs)
- programme aims
- learning outcomes
- structure and content
- teaching approaches
- reflective activities
- action learning
- networking opportunities
- assessment processes

**Comment 8. Relationship to earlier evaluation**

**Response**

We clarified the relationship between the earlier mixed-methods evaluation and the current qualitative analysis.

**Changes made**

Introduction revised:

The programme's design, implementation and mixed-methods evaluation have been reported previously by Doody et al. [13]. That evaluation demonstrated significant improvements in leadership confidence, perceived competence and career development indicators. The present study extends those findings through a stand-alone qualitative analysis exploring participants lived experiences of leadership development and the processes through which they perceived change to occur.

**Comment 9. Research questions**

**Response**

We agree and have added explicit research questions.

**Changes made**

Added at the end of the Introduction:

**Research Questions**

1. How do practice leaders in intellectual disability services experience participation in a Clinical Leadership Programme?
2. How do participants perceive the programme as influencing their leadership identity and everyday leadership practice?

**Comment 10. Theme structure and repetition**

**Response**

We reviewed theme structure carefully. We elected to retain the seven-theme structure as it best reflected the analytic distinctions within participants' accounts. However, we clarified theme boundaries and introduced a conceptual framework (Figure 1) showing relationships among themes organised within four overarching domains.

We also reduced repetition across Findings, Discussion, Implications and Conclusion sections.

**Changes made**

Added:

Although each theme captures a distinct aspect of participants' experiences, considerable overlap and interaction were evident across themes.

Added Figure 1 and explanatory text.

Substantial revisions were also made to reduce repetition in the Discussion, Implications and Conclusion sections.

**Comment 11. Discussion positioning**

**Response**

The Discussion has been revised substantially.

**Changes made**

We strengthened engagement with broader leadership development literature, identified points of agreement and divergence with previous studies and expanded the study's theoretical contribution.

Added:

Unlike leadership development research that emphasises individual competency acquisition, participants placed greater emphasis on identity formation, peer connection and reflective practice as key mechanisms of development.

Added:

The study contributes to leadership development theory by illustrating how leadership identity is shaped through reflection, peer validation, relational learning and organisational context.

**Comment 12. Timing and COVID-19 context**

**Response**

Additional information has been provided regarding interview timing and the COVID-19 context.

**Changes made**

Methods revised:

Participants were interviewed between June and September 2020. The interval between programme completion and interview ranged from approximately 3 to 17 months.

Discussion and Limitations revised:

Data collection occurred during the COVID-19 pandemic, a period characterised by heightened service pressures, workforce strain, altered working arrangements and increased professional isolation.

We also describe the influence of the pandemic on interview delivery and organisational context.

**Comment 13. Terminology and sample description**

**Response**

We agree and have replaced terminology that incorrectly implied an exclusively nursing sample.

**Changes made**

Throughout the manuscript:

intellectual disability nurse leaders

revised to:

practice leaders in intellectual disability services

except where findings specifically refer to nursing participants.

**Comment 14. References**

**Response**

The reference list has been corrected.

**Changes made**

- Duplicate references removed.
- Methodological references replaced with more appropriate qualitative descriptive sources.
- References reviewed for accuracy and attribution.
- Citations supporting claims regarding professional isolation and leadership development were revised.

**Comment 15. Two points of wording**

**15a. Confidence and authority**

**Response**

We revised the wording for clarity.

Original:

Confidence, as described by participants, was not characterised by dominance or authority.

Revised:

Participants described confidence as grounded in self-assurance and professional legitimacy rather than hierarchical authority.

**15b. Qualitative principles**

**Response**

We now explicitly identify the methodological principles informing interpretation.

Added:

Consistent with principles of qualitative description and qualitative content analysis, emphasis was placed on depth, meaning and contextual understanding rather than frequency of occurrence.

**Comment 16. Optional figure**

**Response**

We accepted this suggestion.

**Changes made**

Added **Figure 1: Conceptual Framework Illustrating Relationships Among Themes and Domains Identified in Participants' Accounts of Leadership Development**.

The figure groups seven themes into four overarching domains:

- Developing Leadership Identity
- Relational Leadership
- Leadership in Practice
- Sustaining Development

The figure caption explicitly states that it is not intended to represent a causal or temporal model.

**Reviewer 1**

We thank Reviewer 1 for the positive comments and constructive suggestions.

**Comment 1. Research questions**

**Response:** Addressed through addition of two explicit research questions at the end of the Introduction.

**Comment 2. Programme description**

**Response:** New Clinical Leadership Programme subsection added.

**Comment 3. Recruitment and sample**

**Response:** Recruitment numbers, eligibility, sampling strategy, cohort distribution and sample rationale added.

**Comment 4. Researcher reflexivity**

**Response:** Expanded Data Collection, Data Analysis and Limitations sections.

**Comment 5. Presentation of findings**

**Response:** Additional quotations added. Variation and disconfirming cases incorporated into Findings and Discussion.

**Comment 6. Structure and repetition**

**Response:** Figure 1 introduced and repetition reduced throughout manuscript.

**Comment 7. Timing and COVID-19 context**

**Response:** Time interval (3-17 months) reported and COVID-19 context discussed.

**Comment 8. Causal language**

**Response:** Manuscript reviewed extensively and causal language revised throughout.

**Comment 10. Terminology and references**

**Response:** Terminology corrected and references reviewed and amended.

**Reviewer 2**

We thank Reviewer 2 for recognising the value of the study and providing constructive recommendations.

**Results Comment 1. Visualisation**

**Response:** Figure 1 added.

**Results Comment 2. Supporting quotations**

**Response:** Additional participant quotations incorporated throughout Results, particularly within educational engagement, professional identity and career progression themes.

**Discussion Comment 1. Broader literature**

**Response:** Discussion revised to compare findings with wider leadership development literature.

**Discussion Comment 2. Theoretical contribution**

**Response:** Expanded theoretical contribution added.

**Discussion Comment 3. Positionality**

**Response:** Positionality discussion incorporated into Limitations and Data Analysis sections.

**Reviewer 3**

We thank Reviewer 3 for their positive assessment of the study.

**Comment 1. Longitudinal implication**

**Response:** The term "sustained" and similar wording implying longitudinal evidence were revised or removed throughout.

**Comment 2. Confidence wording**

**Response:** Wording revised to avoid implying a previously established characterisation of confidence.

**Comment 3. Qualitative principles**

**Response:** Methodological principles are now explicitly identified and referenced within the Data Analysis section.

We again thank the Editor and Reviewers for their valuable comments. We believe the revisions have substantially strengthened the manuscript and improved its methodological transparency, analytical rigour and contribution to the leadership development literature.#
