# Supplementary material for: Practice leadership development in intellectual disability services: A qualitative study of practice leaders’ experiences
Source: PLoS One. 2026 Nov 3;21(11):e0351893. doi: 10.1371/journal.pone.0351893 (PMC13730323; doi:10.1371/journal.pone.0351893)
Supplement: Peer review history

# Peer review: Decision Letter 0

**DOI:** 10.1371/journal.pone.0351893.r001

**Authors:** Mia Vogel

10 Sep 2026

Dear Dr. Doody,

Thank you for submitting your manuscript to PLOS One. After careful consideration, we feel that it has merit but does not fully meet PLOS One’s publication criteria as it currently stands. Therefore, we invite you to submit a revised version of the manuscript that addresses the points raised during the review process.

Please submit your revised manuscript by Oct 10 2026 11:59PM. If you will need more time than this to complete your revisions, please reply to this message or contact the journal office at plosone@plos.org. When you're ready to submit your revision, log on to https://www.editorialmanager.com/pone/ and select the 'Submissions Needing Revision' folder to locate your manuscript file.

- A letter that responds to each point raised by the academic editor and reviewer(s). You should upload this letter as a separate file labeled 'Response to Reviewers'.
- A marked-up copy of your manuscript that highlights changes made to the original version. You should upload this as a separate file labeled 'Revised Manuscript with Track Changes'.
- An unmarked version of your revised paper without tracked changes. You should upload this as a separate file labeled 'Manuscript'.

If you would like to make changes to your financial disclosure, please include your updated statement in your cover letter. Guidelines for resubmitting your figure files are available below the reviewer comments at the end of this letter.

If applicable, we recommend that you deposit your laboratory protocols in protocols.io to enhance the reproducibility of your results. Protocols.io assigns your protocol its own identifier (DOI) so that it can be cited independently in the future. For instructions see: https://journals.plos.org/plosone/s/submission-guidelines#loc-laboratory-protocols. Additionally, PLOS One offers an option for publishing peer-reviewed Lab Protocol articles, which describe protocols hosted on protocols.io. Read more information on sharing protocols at https://plos.org/protocols?utm_medium=editorial-email&utm_source=authorletters&utm_campaign=protocols.

As the corresponding author, your ORCID iD is verified in the submission system and will appear in the published article. PLOS supports the use of ORCID, and we encourage all coauthors to register for an ORCID iD and use it as well. Please encourage your coauthors to verify their ORCID iD within the submission system before final acceptance, as unverified ORCID iDs will not appear in the published article. *Only*  the individual author can complete the verification step; PLOS staff *cannot*  verify ORCID iDs on behalf of authors.

We look forward to receiving your revised manuscript.

Kind regards,

Academic Editor

PLOS One

Journal Requirements:

When submitting your revision, we need you to address these additional requirements.

1. Please ensure that your manuscript meets PLOS ONE's style requirements, including those for file naming. The PLOS ONE style templates can be found at

https://journals.plos.org/plosone/s/file?id=wjVg/PLOSOne_formatting_sample_main_body.pdf and

https://journals.plos.org/plosone/s/file?id=ba62/PLOSOne_formatting_sample_title_authors_affiliations.pdf

2. Thank you for stating the following financial disclosure:

"The programme design, delivery, and evaluation were funded by the National Clinical Leadership Centre for Nursing and Midwifery (Office of Nursing and Midwifery Service Directorate) of the Irish Health Service Executive."

Please state what role the funders took in the study.  If the funders had no role, please state: "The funders had no role in study design, data collection and analysis, decision to publish, or preparation of the manuscript."

If this statement is not correct you must amend it as needed.

Please include this amended Role of Funder statement in your cover letter; we will change the online submission form on your behalf.

3. In the online submission form, you indicated that "The data that support the findings of this study are not publicly available due to ethical and confidentiality considerations. Given the qualitative nature of the research and the inclusion of potentially identifiable or sensitive participant information, sharing the dataset could compromise participant privacy. Access to the data is therefore restricted in accordance with the ethical approvals under which this study was conducted. However, limited information may be made available from the corresponding author upon reasonable request, subject to compliance with ethical approval and confidentiality obligations."

All PLOS journals now require all data underlying the findings described in their manuscript to be freely available to other researchers, either 1. In a public repository, 2. Within the manuscript itself, or 3. Uploaded as supplementary information.

This policy applies to all data except where public deposition would breach compliance with the protocol approved by your research ethics board. If your data cannot be made publicly available for ethical or legal reasons (e.g., public availability would compromise patient privacy), please explain your reasons on resubmission and your exemption request will be escalated for approval.

4. If the reviewer comments include a recommendation to cite specific previously published works, please review and evaluate these publications to determine whether they are relevant and should be cited. There is no requirement to cite these works unless the editor has indicated otherwise.

Please review your reference list to ensure that it is complete and correct. If you have cited papers that have been retracted, please include the rationale for doing so in the manuscript text, or remove these references and replace them with relevant current references. Any changes to the reference list should be mentioned in the rebuttal letter that accompanies your revised manuscript. If you need to cite a retracted article, indicate the article’s retracted status in the References list and also include a citation and full reference for the retraction notice.

Additional Editor Comments:

Dear Authors,

Thank you for submitting your manuscript to PLOS ONE. Three reviewers assessed it. Two recommend Minor Revision and one recommends Accept. All three judged the work technically sound and the writing clear, and two independently described the topic as important and underexplored. My decision is Minor Revision.

The reviewers' comments are appended in full and should be addressed individually. Below I consolidate the points that recur across reviews or that I consider necessary for acceptance. Items 1 through 5 are the ones I regard as essential. The remainder will improve the paper but are open to your judgment, provided you explain your reasoning.

1. Researcher positionality and reflexivity. Reviewers 1 and 2 raise this independently, and it is the most important revision. The manuscript states that reflexive discussion took place within the research team (p. 12) but does not report the team's relationship to the program. Please state who conducted the interviews, whether that person contributed to designing, delivering, or evaluating the Clinical Leadership Programme, whether interviewer and participants were known to each other beforehand, and how the team addressed the risk that participants would describe the program favorably to someone connected with it. Please also carry this into the Limitations section, as Reviewer 2 requests, with specific reference to how positionality may have shaped coding and theme development rather than a general acknowledgment.

2. Causal and temporal claims. Reviewer 1 (point 8) and Reviewer 3 (point 1) identify the same issue from different angles. The findings are participant self-reports gathered at one time point. No staff, service users, or family members were interviewed, and leadership practice was not observed. Please review the manuscript throughout and reframe statements that the program produced, strengthened, or caused an outcome as statements about what participants reported, perceived, or attributed. This applies with particular force to claims about staff morale, staff initiative, team functioning, care quality, and leadership sustainability. The sentence on p. 24 beginning "By examining how intellectual disability nurse leaders experienced and enacted a national Clinical Leadership Programme" should be revised, and the word "sustained" removed or bounded to the program period, since it currently implies longitudinal follow-up that the design does not support.

3. Interpretive claims and supporting evidence. Reviewer 2 quotes two passages in the Results that carry substantial interpretive weight but rest entirely on paraphrase, including the material on reframed career trajectories and on reawakened educational identity. Please attach direct participant excerpts to each key claim, or soften the claims to match the evidence actually presented. Reviewer 1 makes a related request for longer quotations that show the context and development of an account rather than a confirming fragment.

4. Analytic variation. Reviewer 1 (point 5) observes that participant experiences are presented as uniformly positive and uniformly similar. Please report where accounts diverged, including participants who did not describe the reported changes, more mixed or negative experiences, and barriers that differed by role, profession, or organizational setting. If convergence was genuine, say so explicitly and describe how you tested for disconfirming cases during analysis. As written, the Results read as confirmation of the program's expected benefits, and the absence of any dissenting account is itself a finding that requires comment.

5. Data Availability Statement. Reviewers disagreed on whether the data underlying the findings are adequately available. Given that the dataset consists of interviews with 14 participants drawn from four deliveries of a single national program, full public deposition may not be appropriate. Please revise the Data Availability Statement to state the specific restriction that applies, identify the institution or committee holding the data, and provide a non-author contact point to which access requests may be directed. A statement that data are available from the authors on request does not satisfy PLOS policy.

6. Recruitment and sampling. Reviewer 1 (point 3) requests detail the manuscript does not currently provide. Please report how many people completed the program across the four deliveries, how many were eligible and invited, how many declined or did not respond, how the 14 were selected, and how they were distributed across deliveries and employing organizations. Please also clarify whether participation was purposive, voluntary and self-selecting, or both, and state the basis on which you judged the sample adequate.

7. Description of the program. Reviewer 1 (point 2) notes that the reader cannot presently identify which program elements might have supported reflection, professional identity, relational leadership, or peer networking. Please add a concise account of duration, content, delivery format, teaching methods, and activities. A citation to the earlier publication is useful but does not substitute, since the manuscript should stand alone.

8. Relationship to the earlier evaluation. Reviewer 3 notes that this study builds on a prior national mixed-methods evaluation of the same program. Please state plainly what was reported in that publication, what is new here, and whether any interviews analyzed in this manuscript were drawn on previously.

9. Research questions. Reviewer 1 (point 1) suggests stating one or two explicit research questions at the end of the Introduction. Hypotheses are not appropriate for an inductive design, but stated questions would clarify the analytic scope and tighten the link between aims, methods, findings, and conclusions. I support this.

10. Theme structure and repetition. Reviewer 1 (point 6) observes overlap among the seven themes, specifically personal transformation, reflection, relational leadership, and extending person-centered values to staff. Please consider a smaller number of themes with subthemes, or justify the current structure by making the boundaries between the seven explicit. There is also repetition across Findings, Discussion, Implications, and Conclusion that could be reduced so that each section does distinct work.

11. Discussion positioning. Reviewer 2 asks that the Discussion compare these findings against the broader literature on leadership development in social and health services, noting points of agreement and disagreement, rather than restating the themes. Reviewer 2 also asks you to state the theoretical gap the study addresses, not only that it complements prior quantitative evaluation. Both would strengthen the section.

12. Timing and the COVID-19 context. Reviewer 1 (point 7) notes that interviews were conducted between June and September 2020 but that the interval between program completion and interview is not reported, despite a later statement that interviews followed shortly after the program. Please specify this interval. Please also comment briefly on whether the pandemic affected program delivery, workload and leadership demands, professional isolation, and the choice of online rather than face to face interviewing. Several themes bear directly on isolation and organizational pressure, so the context is material to interpretation.

13. Terminology and sample description. The sample includes social care leaders, mental health nurses, and intellectual disability nurses, but the Conclusion refers to "intellectual disability nurse leaders," a phrase Reviewer 3 also quotes from p. 24. Please use a term that matches the sample, such as practice leaders in intellectual disability services, or explain why a given conclusion applies only to the nursing subgroup.

14. References. Reviewer 1 reports that references 1 and 6 appear to be the same publication, that reference 14 is not an appropriate methodological source for a qualitative descriptive design, and that reference 12 addresses care quality metrics but is cited in support of claims about professional isolation and limited access to leadership development. Please check these and audit the reference list as a whole for accuracy of attribution.

15. Two points of wording raised by Reviewer 3. On p. 13, the sentence stating that confidence was not characterized by dominance or authority implies a prior characterization that is not established in the text. Please revise or supply the antecedent. On p. 12, the reference to qualitative research principles governing the emphasis on depth over frequency should name the principles or sources relied on.

16. Optional. Reviewer 2 suggests a figure showing the key themes and their interrelations, given that the leadership development process is the study's central concern. I agree this would help readers, though it is a suggestion rather than a condition. If you produce one, it should represent relationships your data support and not imply a sequence or causal ordering that the design cannot establish.

Please supply a point by point response indicating where each change appears in the revised manuscript, and a version with changes marked. Where you decline a suggestion, a brief explanation is sufficient.

I look forward to receiving the revision.

[Note: HTML markup is below. Please do not edit.]

Reviewers' comments:

Reviewer's Responses to Questions

**Comments to the Author**

1. Is the manuscript technically sound, and do the data support the conclusions?

Reviewer #1: Yes

Reviewer #2: Yes

Reviewer #3: Yes

**********

2. Has the statistical analysis been performed appropriately and rigorously?

Reviewer #1: I Don't Know

Reviewer #2: N/A

Reviewer #3: Yes

**********

3. Have the authors made all data underlying the findings in their manuscript fully available?

The PLOS Data policy

Reviewer #1: No

Reviewer #2: Yes

Reviewer #3: Yes

**********

4. Is the manuscript presented in an intelligible fashion and written in standard English?

Reviewer #1: Yes

Reviewer #2: Yes

Reviewer #3: Yes

**********

Reviewer #1: This manuscript addresses an important and underexplored topic and makes a valuable contribution to research on leadership development in intellectual disability services. The article is clear, well-structured and easy to follow. The discussion is especially strong and creates a useful dialogue with the existing literature.

However, several points could be clarified to improve the methodological transparency, the depth of the analysis, and the consistency between the design, results and conclusions.

1. Research question: The general aim can be understood from the end of the introduction, but I suggest stating one or two clear research questions. As this is a qualitative and inductive study, hypotheses are not necessary. However, explicit research questions would help define the analytical scope and clarify the link between the aims, methods, results, and conclusions.

2. Description of the programme: A brief but more detailed description of the Clinical Leadership Programme would be useful. It is difficult to understand which elements may have supported reflection, professional identity, relational leadership, or peer networks without information about its duration, content, teaching methods, delivery format and activities. Even if these details are available in a previous publication, the manuscript should remain understandable on its own.

3. Recruitment and sample: The authors explain that purposive sampling was used and that 14 participants from four programme deliveries were interviewed. However, the recruitment process needs more detail. Please try to briefly clarify: How many people completed the programme, how many were eligible and invited, how many declined or did not respond, how the 14 participants were selected, how they were distributed across the four programme deliveries and organisations and how did you decided that the sample was sufficient.

It would also be useful to clarify whether the sample was mainly purposive or based on voluntary participation and self-selection.

4. Researcher reflexivity: Please include more information about the researchers’ position and their relationship with the programme and participants. For example: who conducted the interviews? Did this person help design or deliver the programme? Did the researchers know the participants before the interviews?

This information is important because participants may have felt pressure to describe the programme positively. The authors should also explain how they reflected on and managed these possible influences during data collection and analysis.

5. Presentation of the findings: The findings are clear and well organised. However, participants’ experiences are often presented as very similar. The analysis would be stronger if it showed more clearly:

• Differences and similarities between participants.

• Less positive or more mixed experiences.

• Participants who did not experience the reported changes.

• Different barriers depending on role, profession, or organisational context.

If the experiences were highly consistent, the authors could explain how different individual experiences converged towards similar findings.

Some longer quotations would also help readers understand the context and development of participants’ experiences. Including contradictory or less positive accounts would strengthen the credibility of the analysis and reduce the impression that the findings mainly confirm the expected benefits of the programme.

6. Structure and repetition: Some of the seven themes appear to overlap, particularly personal transformation, reflection, relational leadership and extending person-centred values to staff. The authors could consider a more integrated structure, with broader themes and subthemes.

There is also some repetition between the findings, discussion, implications, and conclusion. These sections could be shortened so that each has a clearer purpose.

7. Timing and COVID-19 context: The interviews took place between June and September 2020, but the time between programme completion and each interview is not clearly reported. This should be specified, especially because the manuscript later states that interviews took place shortly after the programme.

The authors should also briefly discuss whether the COVID-19 context affected: i) The delivery of the programme, ii) Workload and leadership pressures, iii) Professional isolation and iv) The choice between online and face-to-face interviews.

This is important because several findings relate directly to isolation, overload, and organisational pressure.

8. Causal language: The causal language should be reviewed throughout the manuscript. Because the findings are based on participants’ self-reported perceptions, it would be more accurate to write:

• “Participants reported…”

• “Participants perceived…”

• “Participants attributed these changes to the programme…”

rather than stating directly that the programme produced, strengthened, or caused particular outcomes. This is especially important when discussing staff morale, initiative, team functioning, care quality or leadership sustainability. No staff members, service users, or families were interviewed, and there were no direct observations of leadership practice.

10. Terminology and references: The sample includes social care leaders, mental health nurses and intellectual disability nurses. However, the conclusion refers specifically to “intellectual disability nurse leaders.” This does not represent the full sample. The authors should use “practice leaders in intellectual disability services” or explain why some conclusions are limited to nurses.

The references should also be checked carefully:

1. Reference 14 does not appear to be an appropriate methodological source for a qualitative descriptive design.

2. References 1 and 6 appear to be the same publication.

3. Other citations should be checked for consistency. For example, reference 12 appears to focus on care quality metrics but is used to support claims about professional isolation and limited access to leadership development.

Reviewer #2: Summary

The authors have tackled an important research topic and offer valuable insights for both research and practice. I have several suggestions for the Results and Discussion sections to help the analysis align more closely with the data and existing literature.

Results

1. Visualize the leadership development process. Since the central focus of this study is the leadership development process itself, I recommend the authors design a figure illustrating the key themes and how they interconnect. A visual model would make the shape of this process and the relationships among its components much clearer to readers than a narrative description or table alone.

2. Ground claims in participant quotes. In several places, the Results section summarizes what participants described or expressed without including direct quotes. Every key claim should be supported by an actual excerpt from the interview data. For example:

* "Participants also described how engagement with the programme reframed their understanding of career trajectories within intellectual disability services. Leadership development was no longer conceptualised as a static endpoint associated with managerial appointment, but as a dynamic, evolving process requiring continuous learning. This reframing appeared particularly significant in a sector where opportunities for structured leadership development have historically been limited."

* "Participants expressed a heightened sense of responsibility not only to their own professional growth but to modelling lifelong learning for colleagues and emerging leaders. This theme represents an important dimension of leadership sustainability, highlighting how accessible, relevant leadership programmes can reawaken educational identity and motivate continued professional development. By legitimising learning as an essential component of leadership, the programme contributed to building future capacity within intellectual disability services and reinforcing the long-term viability of leadership roles characterised by complexity, emotional labour, and systemic constraint."

Both passages make substantive interpretive claims but rely entirely on the authors' paraphrase. Adding supporting quotes throughout would strengthen the credibility and transparency of the analysis.

Discussion

1. Situate findings within the broader literature. Rather than restating themes already covered in the Results, the Discussion should explicitly compare this study's leadership development process with the broader literature on leadership development in social services, noting both similarities and differences.

2. Clarify the theoretical contribution. The opening of the Discussion currently frames the study only as complementary to prior quantitative evaluations. The authors should also highlight the specific theoretical gap this study fills and how it contributes to current theorizing on the leadership development process, not just to the existing evidence base.

3. Address researcher positionality in the Limitations section. The Limitations section should reflect on how the researchers' positionality may have shaped the qualitative data analysis.

Reviewer #3: Through the use of inductive qualitative content analysis, this study aims to understand the experiences of individual disability practice leaders as they developed throughout a Clinical Leadership Programme. While initial data was derived from a national mixed-methods analysis of said program conducted within the Republic of Ireland’s intellectual disability services, this study expanded on the results through the completion and analysis of in-depth interviews with individual disability practice leaders that completed this program.

This paper excels in its depth of analysis having a small sample size. The inclusion of direct quotes from in-person interviews allows for increased transparency and translation to concrete, real-world examples of this study’s findings. With qualitative analysis, researchers often have to answer questions about whether their findings can be trusted; in this case, however, researchers did an excellent job of defining their analysis processes up front, as well as keeping an audit trail.

(1) “By examining how intellectual disability nurse leaders experienced and enacted a national Clinical Leadership Programme, the study moves beyond outcome measurement to reveal the mechanisms through which leadership capacity is formed, sustained, and challenged in everyday practice” (p. 24). This statement gives the impression that these are longitudinal findings, especially due to the inclusion of the word “sustained.” Removing this word or making the statement more specific to the time-frame of the programme would resolve the issue.

(1) “Confidence, as described by participants, was not characterised by dominance or authority” (p. 13). This is a minor concern, but the statement gave me the impression that confidence was previously characterized this way.

(2) “Reflexive discussion among the research team helped to challenge assumptions and support confirmability. The qualitative findings are presented to emphasise depth of understanding rather than frequency of responses, in keeping with qualitative research principles” (p. 12). The author does not specify which qualitative research principles they were adhering to; the inclusion of said principles would allow for better understanding of the methodology used in this study.

**********

what does this mean?). If published, this will include your full peer review and any attached files.

If you choose “no”, your identity will remain anonymous but your review may still be made public.

**Do you want your identity to be public for this peer review?**  For information about this choice, including consent withdrawal, please see our Privacy Policy

Reviewer #1: **Yes:**  Leidy Daniela ORTIZ CONRADO

Reviewer #2: No

Reviewer #3: No

**********

[NOTE: If reviewer comments were submitted as an attachment file, they will be attached to this email and accessible via the submission site. Please log into your account, locate the manuscript record, and check for the action link "View Attachments". If this link does not appear, there are no attachment files.]

To ensure your figures meet our technical requirements, please review our figure guidelines: https://journals.plos.org/plosone/s/figures

You may also use PLOS’s free figure tool, NAAS, to help you prepare publication quality figures: https://journals.plos.org/plosone/s/figures#loc-tools-for-figure-preparation.

NAAS will assess whether your figures meet our technical requirements by comparing each figure against our figure specifications.

# Author comment: Author response to Decision Letter 1

**DOI:** 10.1371/journal.pone.0351893.r002

8 Oct 2026

A full response to your feedback has been uploaded we thank you for your time, effort and guidance.

Attachment Submitted filename: Response to Editor and Reviewers.docx

# Editor report: Decision Letter 1

**DOI:** 10.1371/journal.pone.0351893.r003

**Authors:** Mia Vogel

19 Oct 2026

Practice leadership development in intellectual disability services: A qualitative study of practice leaders’ experiences

PONE-D-26-25480R1

Dear Dr. Doody,

We’re pleased to inform you that your manuscript has been judged scientifically suitable for publication and will be formally accepted for publication once it meets all outstanding technical requirements.

Within one week, you’ll receive an e-mail detailing the required amendments. When these have been addressed, you’ll receive a formal acceptance letter and your manuscript will be scheduled for publication.

An invoice will be generated when your article is formally accepted. Please note, if your institution has a publishing partnership with PLOS and your article meets the relevant criteria, all or part of your publication costs will be covered. Please make sure your user information is up-to-date by logging into Editorial Manager at Editorial Manager® and clicking the ‘Update My Information' link at the top of the page. For questions related to billing, please contact billing support.

If your institution or institutions have a press office, please notify them about your upcoming paper to help maximize its impact. If they’ll be preparing press materials, please inform our press team as soon as possible -- no later than 48 hours after receiving the formal acceptance. Your manuscript will remain under strict press embargo until 2 pm Eastern Time on the date of publication. For more information, please contact onepress@plos.org.

Kind regards,

Academic Editor

PLOS One

Additional Editor Comments (optional):

The authors have addressed the reviewer feedback sufficiently.

Reviewers' comments:

# Editor report: Acceptance letter

**DOI:** 10.1371/journal.pone.0351893.r004

**Authors:** Mia Vogel

PONE-D-26-25480R1

PLOS One

Dear Dr. Doody,

I'm pleased to inform you that your manuscript has been deemed suitable for publication in PLOS One. Congratulations! Your manuscript is now being handed over to our production team.

At this stage, our production department will prepare your paper for publication. This includes ensuring the following:

* All references, tables, and figures are properly cited

* All relevant supporting information is included in the manuscript submission,

* There are no issues that prevent the paper from being properly typeset

You will receive further instructions from the production team, including instructions on how to review your proof when it is ready. Please keep in mind that we are working through a large volume of accepted articles, so please give us a few days to review your paper and let you know the next and final steps.

Lastly, if your institution or institutions have a press office, please let them know about your upcoming paper now to help maximize its impact. If they'll be preparing press materials, please inform our press team within the next 48 hours. Your manuscript will remain under strict press embargo until 2 pm Eastern Time on the date of publication. For more information, please contact onepress@plos.org.

You will receive an invoice from PLOS for your publication fee after your manuscript has reached the completed accept phase. If you receive an email requesting payment before acceptance or for any other service, this may be a phishing scheme. Learn how to identify phishing emails and protect your accounts at https://explore.plos.org/phishing.

If we can help with anything else, please email us at customercare@plos.org.

Thank you for submitting your work to PLOS One and supporting open access.

Kind regards,

PLOS One Editorial Office Staff

on behalf of

Dr. Mia T Vogel

Academic Editor

PLOS One
